# Electronic Patient-Reported Outcome Measures in Radiation Oncology: Initial Experience After Workflow Implementation

**DOI:** 10.2196/12345

**Published:** 2019-07-24

**Authors:** Franziska Hauth, Verena Bizu, Rehan App, Heinrich Lautenbacher, Alina Tenev, Michael Bitzer, Nisar Peter Malek, Daniel Zips, Cihan Gani

**Affiliations:** 1 University Hospital Tübingen Department of Radiation Oncology Tübingen Germany; 2 University Hospital Tübingen Section for Information Technology Tübingen Germany; 3 University Hospital Tübingen Internal Medicine I Tübingen Germany; 4 German Cancer Research Center (DKFZ) Heidelberg and German Consortium for Translational Cancer Research (DKTK) Partner Site Tübingen Tübingen Germany

**Keywords:** mHealth, eHealth, radiation oncology, patient reported outcome measures

## Abstract

**Background:**

Mobile health (mHealth) technologies are increasingly used in various medical fields. However, the potential of mHealth to improve patient care in radiotherapy by acquiring electronic patient reported outcome measures (ePROMs) during treatment has been poorly studied so far.

**Objective:**

The aim of this study was to develop and implement a novel Web app (*PROMetheus*) for patients undergoing radiotherapy. Herein, we have reported our experience with a focus on feasibility, patient acceptance, and a correlation of ePROMs with the clinical course of the patients.

**Methods:**

In the period between January and June 2018, 21 patients used PROMetheus to score side effects, symptoms, and quality of life–related parameters during and after their treatment. Items of the Patient Reported Outcome version of the Common Terminology Criteria for Adverse Events (PRO-CTCAE) were chosen based on the primary site of disease, 27 items for head and neck tumors, 21 items for thoracic tumors, and 24 items for pelvic tumors.

**Results:**

In total, 17 out of the 21 patients (81%) regularly submitted ePROMs and more than 2500 data points were acquired. An average of 5.2, 3.5, and 3.3 min was required to complete the head and neck, thorax, and pelvis questionnaires, respectively. ePROMS were able to detect the occurrence of both expected and unexpected side effects during the treatment.

In addition, a gradual increase in the severity of side effects over the course the treatment and their remission afterward could be observed with ePROMs. In total, 9 out of the 17 patients (53%), mostly those with head and neck and thoracic cancers, reported PRO-CTCAE grade III or IV fatigue with severe impairments of activities of daily life.

**Conclusions:**

This study shows the successful implementation of an ePROM system and a high patient acceptance. ePROMs have a great potential to improve patient care in radiotherapy by providing a comprehensive documentation of symptoms and side effects, especially of ones that are otherwise underreported.

## Introduction

### Mobile Health and Patient Reported Outcomes

Mobile health (mHealth) is a rapidly growing field and has, according to the World Health Organization, *the potential to transform the face of health service delivery across the globe* [1]. In oncology, there has been great interest to use mHealth technologies for the acquisition of *patient reported outcome measures* (PROMs), which are then termed electronic PROMs (*ePROMs*) [2-5]. A variety of studies have shown significant benefits of PROMs in terms of improved communication, patient well-being, detection of unrecognized problems, and also, most strikingly, long-term survival [6-9]. ePROMs bring the additional advantage of the immediate availability of PROMs, avoidance of data entry errors, or the possibility of triggering notifications. It has been shown that the data acquired do not differ between a traditional *paper- and pencil-based* assessment and an acquisition via ePROMs [10]. Recently, this was again validated by Matthies et al, who reported highly significant correlations between a paper-based version of the *Functional Assessment of Cancer Therapy—Breast* questionnaire and an electronic version designed for breast cancer patients [11]. For patients undergoing radiotherapy, data regarding the use of ePROMs are sparse despite surveys showing a considerable interest to use mobile technologies in clinical practice, both on the caregiver and patient side [12,13].

### Electronic Patient Reported Outcome Measures in Radiotherapy

In radiotherapy, where the majority of treatments are performed in an outpatient setting, ePROMs might be a useful tool to monitor acute toxicities during therapy and shortly afterward, as well as late toxicities. Furthermore, signs of disease recurrence or progression might be detected at an earlier stage. We developed a Web-based application, *PROMetheus*, that allows patients to submit ePROMs over the internet to the treating team. We hypothesized that ePROMs will be well accepted by our patients and provide a complete and comprehensive documentation of side effects and quality of life (QoL)–related parameters during radiotherapy. In this study, we report our initial experience with a focus on feasibility, patient acceptance, and a correlation of ePROMs with the clinical course of the patients.

## Methods

### Participants and Recruitment Process

In the period between January 2018 and June 2018, 21 patients used PROMetheus to score symptoms and QoL-related parameters. Patients who had provided an email address in their demographic data were approached by a physician before treatment or latest during the first week of treatment and asked if they were interested to use PROMetheus. All the patients who were offered to use PROMetheus provided consent. If requested, the first scoring was completed under supervision. After this, the patients were instructed to complete a Web-based questionnaire whenever desired but at least once weekly. A reminder to complete the questionnaire was sent once a week via email. In general, patients were approached by the treating physician whenever Patient Reported Outcomes-Common Terminology Criteria for Adverse Event (PRO-CTCAE) grade IV toxicity was reported on the Web or when a 2-point increase from grade I to grade III was observed. ePROMs were reviewed daily by the principal investigator of the study (CG). We had defined that no medical intervention or treatment would be initiated solely based on ePROMs without a confirmatory interaction of the treating physician with the patient. Irrespective of the ePROM submission, patients had weekly consultations with the treating physician to assess toxicity according to our institutional standard. If a patient was admitted for inpatient treatment, the patient was asked to continue the ePROM submission and email reminders were continued. Free Wi-Fi access was offered to all inpatients in the study. This study was approved by the institutional review board of the medical faculty in Tuebingen, Germany (approval number: 421/2018B02).

### Technology Platform Development

**Figure 1:**
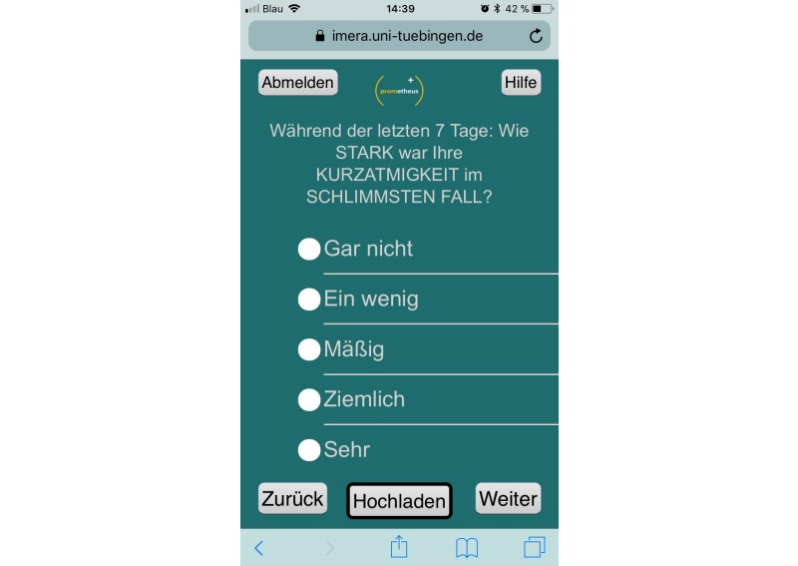
Screenshot of PROMetheus during usage (functions of the buttons: top left=sign out, top right=help, bottom left=back, bottom center=upload all items answered so far, and bottom right=next). The question displayed assesses the severity of shortness of breath with a 5-tier scale from not at all to very.

PROMetheus was developed as a progressive Web application using HTML5, CSS3, and JavaScript and is accessible through browsers on all internet-compatible devices such as smartphones, tablets, or computers. Patients log in with an alphanumeric pseudonym and a password and are then immediately forwarded to the first item. Figure 1 depicts a screenshot of PROMetheus. PROMetheus is built on the *Integrated Mobile Health Research Platform* (IMeRa), which facilitates the submission of data from the patient to the hospital network over the internet. IMeRa provides a Secure Sockets Layer–encrypted Web service for Web applications. Via this Web service, the patients’ answers are transmitted in the *Mobile Data Repository for Research* of the IMeRa platform (based on an Oracle Relational Database Management System [RDBMS]). The IMeRa platform also provides the physician with a Web-based data browser where patient data can be selected, displayed, and exported for further statistical evaluations.

### Structure of the Questionnaires

The questionnaires fed into PROMetheus were based on the certified German translation of the PRO-CTCAE developed by the National Cancer Institute [14]. In short, the questions assess severity, frequency, and impact on activities of daily life (ADL) on a 5-tier scale from *none* to *very severe* or from *never* to *almost always* based on the average of the last 7 days. Questions referring to the severity or the impact on ADLs of an item were automatically skipped if the given symptom was not present. We defined 3 different sets of questions for the included treatment categories: head and neck (up to 27 questions), thoracic (up to 21 questions), and abdominal (up to 24 questions) tumors, respectively (Multimedia Appendices 1-3). For head and neck cancer patients, we individually developed 3 items that assessed pain and swallowing. Patients had the opportunity to discontinue and submit the completed items at any point; however, it was not possible to skip questions.

### Data Analysis

Regarding *patient acceptance*, we defined ePROMs as accepted by a patient if the ePROMs were submitted at 5 time points at least, which approximately corresponds to weekly submissions. For descriptive data, median values and interquartile (25th to 75th quantile) ranges (IQR) are reported. For statistical analysis SPSS version 25 (IBM) and Microsoft Excel were used.

## Results

### Patient and Treatment Characteristics

Patient characteristics are shown in Table 1. All the patients (n=21) completed radiotherapy with a median treatment dose of 60 Gy (IQR 50.4 Gy to 64 Gy). Patients received either radiotherapy alone (5 patients) or radiochemotherapy (16 patients). In total, 12 patients received the concomitant systemic treatment in a preplanned inpatient setting. In most cases, this inpatient treatment took place during the first and fifth week of treatment, whereas treatment was conducted in an outpatient setting on the remaining days. No admissions for inpatient treatment occurred because of toxicity.

**Table 1:** Patient and treatment characteristics.

Characteristics		Value
Total number of patients, N (%)		21 (100)
**Sex, n (%)**		
	Male	14 (67)
	Female	7 (33)
Age (years), median (IQR^a^)		59.4 (51.5-66.5)
**Treatment, n (%)**		
	Radiotherapy	5 (24)
	Radiochemotherapy	16 (76)
Radiotherapy dose (Gray), median (IQR)		60 (50.4-64)
**Primary site of cancer, n (%)**		
	Pelvic	10 (48)
	Thoracic	5 (24)
	Head and neck	4 (19)
	Upper gastrointestinal	2 (10)
**Stage^b^, n (%)**		
	I	1 (5)
	II	5 (24)
	III	13 (62)
	IV	1 (5)

^a^IQR: interquartile range.

^b^One patient with a tumor of an unknown primary site was excluded.

### Feasibility and Patient Acceptance

**Figure 2:**
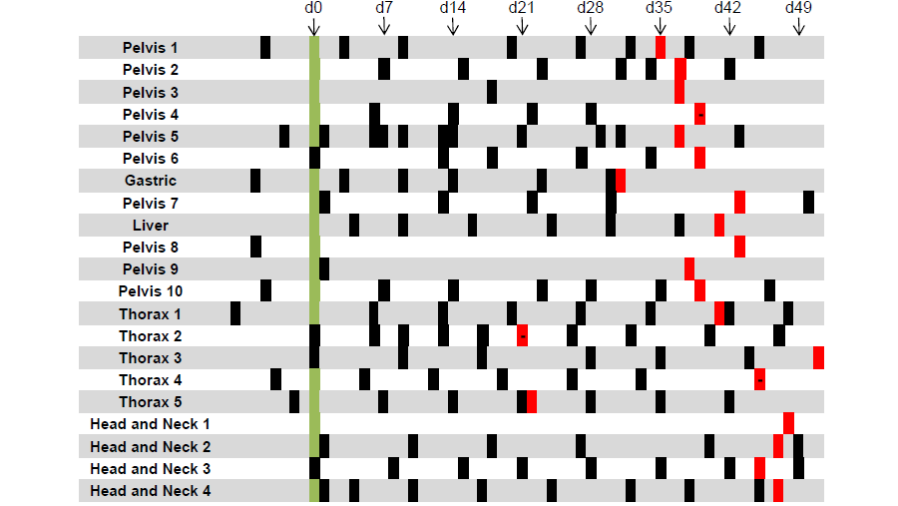
Time points with electronic patient-reported outcome measure (ePROM) submissions of the 21 patients in our cohort. d0 (green) indicates the day of the first treatment, time points filled in red indicate the last day of treatment. Red bars with a dash indicate ePROM submission on the last day of treatment. d: day.

ePROM acquisition with PROMetheus was feasible. None of the patients reported any technical issues that prevented the submission of ePROMs or problems in understanding the usage of PROMetheus. In terms of patient acceptance, 17 out of 21 patients (81%) regularly submitted ePROM data during treatment, with a median of 6 (IQR 4 to 8) submissions (Figure 2).

The patients required a median of 5.45 min (IQR 4.65 to 5.91 min), 2.39 min (IQR 2.38 to 4.78 min), and 2.95 min (IQR 2.72 to 3.36 min) for the completion of the head and neck, thorax, and abdominal questionnaires, respectively. By including the submissions after the end of treatment, we were able to collect more than 2500 data points in these 21 patients. In all the submitted questionnaires, all items had been completed.

### Clinical Examples of Electronic Patient-Reported Outcome Measures Assessed During and After Treatment

**Figure 3:**
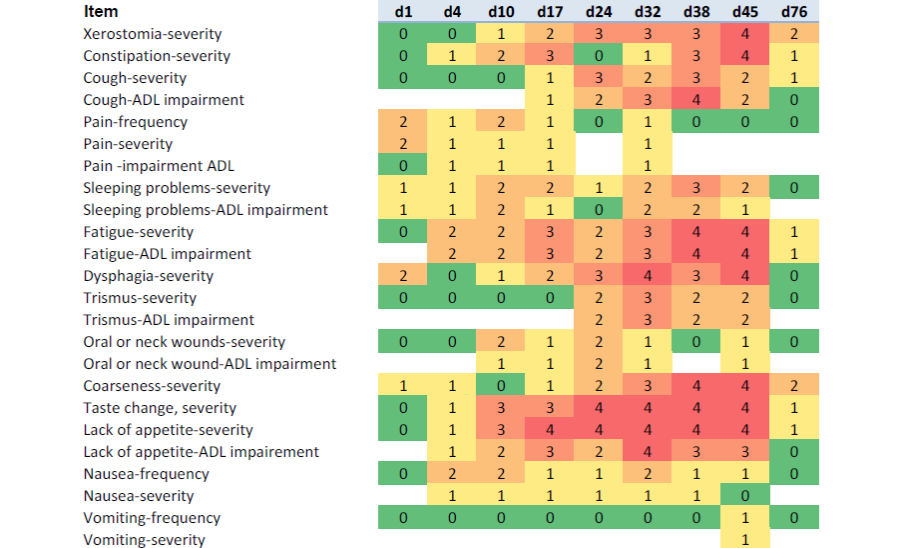
Example of the course of electronic patient reported outcome measures in a 75-year-old patient who underwent definitive radiochemotherapy for locally advanced head and neck cancer. Day 45 constitutes the last assessment during therapy; d76 was approximately 3 weeks after the end of treatment. d: day; ADL: activities of daily life.

Figure 3 shows an example of a 75-year-old patient who was treated for a locally advanced tumor at the base of the tongue (cT2 cN2b cM0) using definitive radiotherapy with 70 Gy and concomitant chemotherapy with weekly Cisplatin (40 mg/m²). A percutaneous endoscopic gastrostomy (PEG) tube was placed 2 days before the initiation of the treatment. Treatment toxicity and impairment of ADL continuously increased with treatment time. Taste changes and lack of appetite were the PROMs to reach grade III or higher. Three weeks after the end of treatment, all high-grade toxicities had resolved.

ePROMs from 2 other patients with rectal cancer and gastric lymphoma are shown in Multimedia Appendices 4 and 5. The first patient (male, aged 50 years) was treated using preoperative radiochemotherapy (50.4 Gy) with concomitant 5-fluorouracil during the first and fifth week for locally advanced rectal cancer. The only PRO-CTCAE grade IV toxicity during the treatment was the *severity of vomiting* reported on the third day of radiochemotherapy. Further investigation revealed a norovirus as the cause of this toxicity. The other patient was a 55-year-old female diagnosed with mucosa-associated lymphoma tissue (MALT) lymphoma of the stomach. Treatment using radiotherapy with a total dose of 39.6 Gy was planned, and prophylactic intravenous antiemetics were offered. On the basis of the patient’s request, only oral antiemetics were prescribed and ePROMs were assessed during the treatment. No nausea or vomiting of PRO-CTCAE grade III or IV was scored during the treatment. Instead, fatigue and *sleeping problems* were the highest scored items. The patient completed the treatment without any intravenous treatment.

### Fatigue as the Most Frequently Reported Single Item

With regard to fatigue, 9 out of the 17 patients (53%) who regularly submitted ePROMs reported PRO-CTCAE grade III or IV fatigue at the end of the treatment; in all cases, impairment of ADLs was scored as PRO-CTCAE grade III or IV as well. All the patients except one with head and neck or thoracic cancer had PRO-CTCAE grade III or IV fatigue, whereas only 1 patient with a pelvic primary had scored high-grade fatigue.

## Discussion

### Principal Findings, Strengths, and Limitations

This study is one of the first to report ePROMs for the assessment of treatment toxicity and QoL aspects in patients undergoing radiotherapy. We observed high acceptance with over 80% of the patients regularly submitting complete ePROM datasets. We believe that this was facilitated by the very simple setup of PROMetheus and the weekly reminders for the completion of the questionnaires, which confirms the results of a patient survey conducted by El Shafie et al who reported a great interest in patients to use mobile technologies during radiotherapy and thereafter [12]. The potential benefits of ePROMs are manifold. First, concerns regarding a potential underreporting of toxicities in clinical trials have repeatedly been expressed [15]. Usually, toxicity during treatment is evaluated by a physician at regular intervals and entered into the patient’s chart, either already graded according to a standard scoring system or as descriptive text. In case of the latter, this information needs to be *translated* into a grading system (potentially by a third person) if a graded score is required, for instance, for entry into a research database. All these individual steps are prone to errors or loss of crucial information [16,17]. With ePROMs, this information is immediately available as digital and parametric data. Furthermore, patients can go through the items at home or in the waiting area, without the stress associated with a doctor’s visit. It has been suggested in many studies that self-reporting increases patient contentedness and potentially enables physicians to recognize adverse events at an earlier time point [18,19]. Clearly, a limitation of this study is the sample size, which does not permit detailed subgroup analyses. The rationale for the sample size was to evaluate the feasibility and patient acceptance at an early time point before moving forward to large-scale randomized trials. Even though the patient cohort in this study is small, it represents patients with various tumor entities and age groups.

### Further Benefits of Electronic Patient-Reported Outcome Measures

Self-reporting may also improve communication between physicians and patients, as investigated by Velikova et al who showed in a randomized trial that regular assessment of QoL improved patient-physician communication even resulting in better emotional functioning and overall QoL [7]. We made the experience that offering a Web-based PROM solution lowered the patients’ threshold to contact the treating physician, for instance, via email when symptoms occurred that were not assessed by the predefined questionnaires. For example, 1 patient with nonsmall cell lung cancer and receiving definitive radiochemotherapy developed severe lower back pain only a few weeks after the end of treatment. As no PRO-CTCAE item in our questionnaires assessed pain outside the treated area, the patient submitted this symptom via email, which prompted a face to face assessment of pain characteristics, physical examination, and, subsequently, a computed tomography within a few days, confirming the suspected metastatic disease and the swift initiation of systemic treatment. It appears likely that the early detection of disease progression, as in our exemplary case, played a crucial role in a recently published study of ePROMs in the care of cancer patients undergoing palliative chemotherapy. In this randomized trial of 766 patients, in which the sole intervention was the inclusion of a Web-based ePROM platform in the experimental arm, a 5-month benefit in overall survival was seen [8].

We consider ePROMs as a very useful complement to face-to-face patient-physician interaction and not a replacement. First, according to a recent survey, a considerable number of patients would refuse ePROM usage because of the wish for personal contact with the treating physician [13]. Second, and most importantly, self-reported high-grade toxicities and symptoms scored with ePROMs need clinical validation before any diagnostic or therapeutic intervention is initiated. This is well reflected by the mentioned case of a rectal cancer patient whose severe diarrhea and vomiting during the first week of treatment turned out to have a viral origin rather than being a treatment-related side effect (Multimedia Appendix 4). As we approached patients who had provided an email address during the registration process, our cohort has to be considered a positive selection in terms of computer experience. This is an important aspect, as Basch et al showed a correlation between Web usage before enrollment and log-in times during data acquisition [16]. On the contrary, the benefits of ePROM usage in terms of survival and frequency of emergency room visits have been shown to be independent of computer experience [19]. It is likely that the technological progress and the increasing incorporation of the internet into our daily life will soon limit the patient cohort that is either not able to or not willing to use ePROMs.

### Content Validity of the Selected Items

Content validity is an important aspect in the context of PROMs. In an interview-based study among radiotherapy patients—published before we designed our questionnaires—Sandler et al found that all except 5 frequently reported toxicities were covered by PRO-CTCAE items. In total, 3 of these items were seen in patients with head and neck cancers (mucus production, oral pain, and pain when swallowing) [20]. Indeed, when we compiled the items for the 3 treatment sites, we saw the need to add additional items that assessed PEG usage and pain medication intake.

### The Challenge of Big Data

A major challenge faced when implementing ePROMs in clinical practice is the huge amount of data, which needs to be reviewed. Notification systems that inform the treating team of either uncommon symptoms or critical severity of toxicity before meeting the patient need to be implemented. Defining these thresholds for all items already constitutes a challenge of its own and warrants further research [21-23].

### Electronic Patient-Reported Outcome Measures and Cancer-Related Fatigue

It is well known that cancer-related fatigue (CRF) is an underreported symptom in patients undergoing treatment [24]. Conventional symptom assessment by clinicians has been shown to diagnose *subjective* symptoms, such as fatigue, less frequently than self-reporting using PROM items [25]. Indeed, almost all patients who received treatment of the head and neck or thoracic tumors self-reported a severe level of fatigue. Even more important is that all these patients considered their fatigue to be impairing their activities to a high degree. Several groups were able to show that exercise may have an impact on the severity of CRF [26]. A meta-analysis by Tomlinson et al reported a positive effect of exercise on fatigue as well as depression and sleep disturbance. At our department, we are currently evaluating the potential of commercially available activity trackers to tackle CRF and to implement ePROMs as a tool to monitor CRF [27,28].

### Optimization of Normal Tissue Complication Probability Models Through Electronic Patient-Reported Outcome Measures

The large amount of data that accumulate when PROMs are implanted into the clinical workflow can have a much broader use than the sole monitoring of PROMs. Weighing the likelihood of acute and long-term toxicities against tumor control probabilities is the daily routine in radiotherapy. Normal Tissue Complication Probability models have been established to estimate the likelihood of a specific toxicity [29]. The majority of these models are based on clinician-assessed endpoints with the associated limitations discussed earlier. One can envision that PROM data could be used to further refine these models and improve their ability to predict long-term toxicities, similar to an approach presented by Miften et al [30]. Here, the selection of the adequate items is crucial, and a recent review has shown that only a minority of PROM-based models is accurate or can be generalized on external validation [31].

In conclusion, our results show that the implementation of an ePROM system for the assessment of treatment side effects and QoL during radiotherapy is feasible and well accepted by the patients. We, therefore, consider ePROMs as a very useful tool to complement face-to-face patient-physician interaction. However, randomized trials will have to prove whether a measurable benefit through ePROMs in terms of QoL, side effects, or even survival can be achieved [32]. Furthermore, the development of strategies to handle the large amounts of data is among the major challenges that need to be addressed in the future.

## Appendix

Multimedia Appendix 1 Questionnaire for patients with head and neck cancer.

Multimedia Appendix 2 Questionnaire for patients with thoracic tumors.

Multimedia Appendix 3 Questionnaire for patients with abdominal or pelvic cancer.

Multimedia Appendix 4 Electronic patient-reported outcome measures of a 50-year-old male patient who received preoperative radiochemotherapy for locally advanced rectal cancer are depicted. The only grade IV score was the severity of vomiting at a very early stage of treatment. After a medical history, this turned out to be caused by a noroviral infection.

Multimedia Appendix 5 Electronic patient-reported outcome measures of a 55-year-old female patient who underwent radiotherapy of the stomach for mucosa-associated lymphoma tissue lymphoma. Although nausea or vomiting were not an issue until the end of the treatment, fatigue and sleeping problems were the predominant symptoms during the treatment.

## Abbreviations

ADL: activities of daily life
CRF: cancer-related fatigue
ePROM: electronic patient-reported outcome measure
IMeRa: Integrated Mobile Health Research Platform
IQR: interquartile range
mHealth: mobile health
PEG: percutaneous endoscopic gastrostomy
PRO-CTCAE: Patient Reported Outcomes-Common Terminology Criteria for Adverse Events
QoL: quality of life
