# Supplementary material for: Electronic Patient-Reported Outcome Measures in Radiation Oncology: Initial Experience After Workflow Implementation
Source: JMIR Mhealth Uhealth. 2019 Jul 24;7(7):e12345. doi: 10.2196/12345 (PMC6685133; doi:10.2196/12345)
Supplement: Multimedia Appendix 3 [file mhealth_v7i7e12345_app3.pdf]

NCI PRO-CTCAE™ ITEMS

Item Library Version 1.0
English
Form created on 5 September 2018

As individuals go through treatment for their cancer they sometimes experience different symptoms and side effects. For each question, please check or mark an ☒ in the one box that best describes your experiences over the past 7 days…

1.	In the last 7 days, what was the SEVERITY of your DECREASED APPETITE at its WORST?				
	○ None	○ Mild	○ Moderate	○ Severe	○ Very severe
	In the last 7 days, how much did DECREASED APPETITE INTERFERE with your usual or daily activities?				
	○ Not at all	○ A little bit	○ Somewhat	○ Quite a bit	○ Very much

2.	In the last 7 days, how OFTEN did you have NAUSEA?				
	○ Never	○ Rarely	○ Occasionally	○ Frequently	○ Almost constantly
	In the last 7 days, what was the SEVERITY of your NAUSEA at its WORST?				
	○ None	○ Mild	○ Moderate	○ Severe	○ Very severe

3.	In the last 7 days, how OFTEN did you have VOMITING?				
	○ Never	○ Rarely	○ Occasionally	○ Frequently	○ Almost constantly
	In the last 7 days, what was the SEVERITY of your VOMITING at its WORST?				
	○ None	○ Mild	○ Moderate	○ Severe	○ Very severe

4.	In the last 7 days, what was the SEVERITY of your CONSTIPATION at its WORST?				
	○ None	○ Mild	○ Moderate	○ Severe	○ Very severe

5.	In the last 7 days, how OFTEN did you have LOOSE OR WATERY STOOLS (DIARRHEA/DIARRHOEA)?				
	○ Never	○ Rarely	○ Occasionally	○ Frequently	○ Almost constantly

The PRO-CTCAE™ items and information herein were developed by the NATIONAL CANCER INSTITUTE at the NATIONAL INSTITUTES OF HEALTH, in Bethesda, Maryland, U.S.A. Use of the PRO-CTCAE™ is subject to NCI's Terms of Use.

6.	In the last 7 days, how OFTEN did you have PAIN IN THE ABDOMEN (BELLY AREA)?				
	○ Never	○ Rarely	○ Occasionally	○ Frequently	○ Almost constantly
	In the last 7 days, what was the SEVERITY of your PAIN IN THE ABDOMEN (BELLY AREA) at its WORST?				
	○ None	○ Mild	○ Moderate	○ Severe	○ Very severe
	In the last 7 days, how much did PAIN IN THE ABDOMEN (BELLY AREA) INTERFERE with your usual or daily activities?				
	○ Not at all	○ A little bit	○ Somewhat	○ Quite a bit	○ Very much

7.	In the last 7 days, how OFTEN did you LOSE CONTROL OF BOWEL MOVEMENTS?				
	○ Never	○ Rarely	○ Occasionally	○ Frequently	○ Almost constantly
	In the last 7 days, how much did LOSS OF CONTROL OF BOWEL MOVEMENTS INTERFERE with your usual or daily activities?				
	○ Not at all	○ A little bit	○ Somewhat	○ Quite a bit	○ Very much

8.	In the last 7 days, how OFTEN did you have PAIN?				
	○ Never	○ Rarely	○ Occasionally	○ Frequently	○ Almost constantly
	In the last 7 days, what was the SEVERITY of your PAIN at its WORST?				
	○ None	○ Mild	○ Moderate	○ Severe	○ Very severe
	In the last 7 days, how much did PAIN INTERFERE with your usual or daily activities?				
	○ Not at all	○ A little bit	○ Somewhat	○ Quite a bit	○ Very much

9.	In the last 7 days, what was the SEVERITY of your INSOMNIA (INCLUDING DIFFICULTY FALLING ASLEEP, STAYING ASLEEP, OR WAKING UP EARLY) at its WORST?				
	○ None	○ Mild	○ Moderate	○ Severe	○ Very severe
	In the last 7 days, how much did INSOMNIA (INCLUDING DIFFICULTY FALLING ASLEEP, STAYING ASLEEP, OR WAKING UP EARLY) INTERFERE with your usual or daily activities?				
	○ Not at all	○ A little bit	○ Somewhat	○ Quite a bit	○ Very much

The PRO-CTCAE™ items and information herein were developed by the NATIONAL CANCER INSTITUTE at the NATIONAL INSTITUTES OF HEALTH, in Bethesda, Maryland, U.S.A. Use of the PRO-CTCAE™ is subject to NCI's Terms of Use.

NCI PRO-CTCAE™ ITEMS

Item Library Version 1.0
English
Form created on 5 September 2018

10.	In the last 7 days, what was the SEVERITY of your FATIGUE, TIREDNESS, OR LACK OF ENERGY at its WORST?				
	○ None	○ Mild	○ Moderate	○ Severe	○ Very severe
	In the last 7 days, how much did FATIGUE, TIREDNESS, OR LACK OF ENERGY INTERFERE with your usual or daily activities?				
	○ Not at all	○ A little bit	○ Somewhat	○ Quite a bit	○ Very much

11.	In the last 7 days, what was the SEVERITY of your PAIN OR BURNING WITH URINATION at its WORST?				
	○ None	○ Mild	○ Moderate	○ Severe	○ Very severe

12.	In the last 7 days, how OFTEN did you feel an URGE TO URINATE ALL OF A SUDDEN?				
	○ Never	○ Rarely	○ Occasionally	○ Frequently	○ Almost constantly
	In the last 7 days, how much did SUDDEN URGES TO URINATE INTERFERE with your usual or daily activities?				
	○ Not at all	○ A little bit	○ Somewhat	○ Quite a bit	○ Very much

13.	In the last 7 days, were there times when you had to URINATE FREQUENTLY?				
	○ Never	○ Rarely	○ Occasionally	○ Frequently	○ Almost constantly
	In the last 7 days, how much did FREQUENT URINATION INTERFERE with your usual or daily activities?				
	○ Not at all	○ A little bit	○ Somewhat	○ Quite a bit	○ Very much

14.	In the last 7 days, how OFTEN did you have LOSS OF CONTROL OF URINE (LEAKAGE)?				
	○ Never	○ Rarely	○ Occasionally	○ Frequently	○ Almost constantly
	In the last 7 days, how much did LOSS OF CONTROL OF URINE (LEAKAGE) INTERFERE with your usual or daily activities?				
	○ Not at all	○ A little bit	○ Somewhat	○ Quite a bit	○ Very much

The PRO-CTCAE™ items and information herein were developed by the NATIONAL CANCER INSTITUTE at the NATIONAL INSTITUTES OF HEALTH, in Bethesda, Maryland, U.S.A. Use of the PRO-CTCAE™ is subject to NCI's Terms of Use.

NCI PRO-CTCAE™ ITEMS

Item Library Version 1.0
English
Form created on 5 September 2018

Do you have any other symptoms that you wish to report?	
○ Yes	○ No

Please list any other symptoms:

1.	In the last 7 days, what was the SEVERITY of this symptom at its WORST?				
	○ None	○ Mild	○ Moderate	○ Severe	○ Very severe
2.	In the last 7 days, what was the SEVERITY of this symptom at its WORST?				
	○ None	○ Mild	○ Moderate	○ Severe	○ Very severe
3.	In the last 7 days, what was the SEVERITY of this symptom at its WORST?				
	○ None	○ Mild	○ Moderate	○ Severe	○ Very severe
4.	In the last 7 days, what was the SEVERITY of this symptom at its WORST?				
	○ None	○ Mild	○ Moderate	○ Severe	○ Very severe
5.	In the last 7 days, what was the SEVERITY of this symptom at its WORST?				
	○ None	○ Mild	○ Moderate	○ Severe	○ Very severe

The PRO-CTCAE™ items and information herein were developed by the NATIONAL CANCER INSTITUTE at the NATIONAL INSTITUTES OF HEALTH, in Bethesda, Maryland, U.S.A. Use of the PRO-CTCAE™ is subject to NCI's Terms of Use.
