# Supplementary material for: Electronic Patient-Reported Outcome Measures in Radiation Oncology: Initial Experience After Workflow Implementation
Source: JMIR Mhealth Uhealth. 2019 Jul 24;7(7):e12345. doi: 10.2196/12345 (PMC6685133; doi:10.2196/12345)
Supplement: Multimedia Appendix 4 [file mhealth_v7i7e12345_app4.pdf]

	Days after start of treatment								
**Item**	**-d6**	**d3**	**d9**	**d20**	**d27**	**d32**	**d38**	**d45**	**d78**
Lack of appetite - severity	2	2	0	0	0	1	0	0	0
Lack of appetite - ADL impairement	0	0				0			
Dysuria - Severity	0	0	0	0	0	0	0	0	0
Urine Urge -Frequency	0	0	0	0	0	0	0	0	0
Urine urge - ADL impairment									
Urinary frequency	0	1	1	0	0	0	0	0	0
Urinary frequency - ADL impairment		0	0						
Urinary incontinance - frequency	0	0	0	0	0	0	0	0	0
Urinary incontinance - ADL impairment									
Nausea - Frequency	0	1	0	0	0	1	1	0	0
Nausea - Severity		2				1	1		
Vomiting - frequency	0	1	0	0	0	0	0	0	0
Vomiting - severity		4							
Constipation - severity	0	0	0	0	1	0	0	1	0
Diarrhea frequency	3	1	2	1	0	0	0	0	0
Abdominal pain - frequency	1	1	0	0	0	0	0	0	0
Abdominal pain - severity	1	2							
Abdominal pain - ADL impairment	0	1							
Fecal Incontinence - frequency	1	0	0	1	0	0	0	0	0
Fecal Incontinence - ADL impairment	1			0					
Sleeping problems - severity	3	1	2	1	2	1	1	0	1
Sleeping problems - ADL impairment	2	0	0	0	0	0	0		0
Fatigue - severity	2	1	0	1	1	1	1	1	1
Fatigue - ADL impairment	2	1		1	0	0	0	0	1
