# Supplementary material for: Electronic Patient-Reported Outcome Measures in Radiation Oncology: Initial Experience After Workflow Implementation
Source: JMIR Mhealth Uhealth. 2019 Jul 24;7(7):e12345. doi: 10.2196/12345 (PMC6685133; doi:10.2196/12345)
Supplement: Multimedia Appendix 5 [file mhealth_v7i7e12345_app5.pdf]

	Days after start of treatment					
**Item**	**-d7**	**d3**	**d9**	**d14**	**d23**	**d30**
Lack of appetite - severity	0	0	0	0	0	0
Lack of appetite - ADL impairement						
Dysuria - Severity	0	0	0	0	0	0
Urine Urge -Frequency	0	1	1	1	0	0
Urine urge - ADL impairment		0	0	0		
Urinary frequency	0	2	2	1	1	0
Urinary frequency - ADL impairment		1	0	0	0	
Urinary incontinance - frequency	0	0	0	0	0	0
Urinary incontinance - ADL impairment						
Nausea - Frequency	1	1	2	1	1	1
Nausea - Severity	1	1	2	1	1	1
Vomiting - frequency	0	0	0	0	0	0
Vomiting - severity						
Obstipation - severity	0	1	1	2	1	2
Diarrhea frequency	0	0	0	0	0	0
Abdominal pain - frequency	0	1	2	1	0	0
Abdominal pain - severity		1	2	1		
Abdominal pain - ADL impairment		1	2	1		
Fecal Incontinence - frequency	0	0	0	0	0	0
Fecal Incontinence - ADL impairment						
Sleeping problems - severity	2	1	2	2	1	3
Sleeping problems - ADL impairment	1	0	1	1	2	3
Fatigue - severity	1	1	2	2	3	3
Fatigue - ADL impairment	1	1	2	2	3	3
